# Burnout syndrome among healthcare professionals in intensive care units: a cross-sectional population-based study

**DOI:** 10.5935/0103-507X.20200036

**Published:** 2020

**Authors:** Maria Emília Miranda Alvares, Erika Barbara Abreu Fonseca Thomaz, Zeni Carvalho Lamy, Rachel Vilela de Abreu Haickel Nina, Marina Uchoa Lopes Pereira, João Batista Santos Garcia

**Affiliations:** 1 Departamento de Saúde Pública, Universidade Federal do Maranhão - São Luís (MA), Brasil.; 2 Unidade do Sistema Cardiovascular, Hospital Universitário, Universidade Federal do Maranhão - São Luís (MA), Brasil.; 3 Serviço de Dor e Cuidados Paliativos, Universidade Federal do Maranhão - São Luís (MA), Brasil.

**Keywords:** Burnout, psychological, Critical care, Health personnel, Stress, psychological, Intensive care units, Esgotamento psicológico, Cuidados críticos, Pessoal de saúde, Estresse psicológico, Unidades de terapia intensiva

## Abstract

**Objective:**

To assess the prevalence of and factors associated with Burnout syndrome among intensive care unit professionals.

**Methods:**

In this cross-sectional population-based study, a questionnaire assessing sociodemographic, behavioral, and occupational data was administered to 241 nurses and physicians working in 17 public intensive care units in São Luis (MA), Brazil. The Maslach Burnout Inventory - Human Services Survey was used to identify Burnout syndrome based on Maslach’s and Grunfeld’s criteria. The prevalence of each dimension of the syndrome was estimated with a 95% confidence interval. Associations were estimated by the odds ratios via multiple logistic regression analyses (α = 5%).

**Results:**

The prevalence of Burnout syndrome was 0.41% (0.01 - 2.29) according to Maslach’s criteria and 36.9% (30.82 - 43.36) according to Grunfeld’s criteria. Infant intensive care unit professionals were more likely to develop emotional exhaustion than other intensive care professionals (OR = 3.16). Respondents over the age of 35 were less likely to develop emotional exhaustion (OR = 0.32) and depersonalization (OR = 0.06). Longer working hours in intensive care units were associated with a reduced sense of personal accomplishment (OR = 1.13). Among nurses, males had a lower sense of professional accomplishment, and not exercising regularly was associated with more emotional exhaustion and less depersonalization. Among physicians, working in infant and cardiology intensive care units made them less likely to have a reduced sense of personal accomplishment, and physicians without a postgraduate degree who worked in intensive care units had a higher chance of having a lower sense of personal accomplishment.

**Conclusion:**

This study demonstrated the low prevalence of Burnout syndrome. Most of the professionals reported low levels for each dimension of Burnout, including low levels of emotional exhaustion, low levels of depersonalization, and a lower likelihood of having a reduced sense of personal accomplishment. Nurses and physicians have different characteristics associated with Burnout syndrome.

## Abstract (pt)

**Objetivo:**

Avaliar a prevalência e os fatores associados com a síndrome de *burnout* em profissionais que atuam em unidade de terapia intensiva.

**Métodos:**

Estudo transversal com base populacional. Aplicou-se um questionário sociodemográfico, comportamental e ocupacional a 241 enfermeiros e médicos atuantes em 17 unidades de terapia intensiva públicas na cidade de São Luís (MA). Utilizou-se o *Maslach Burnout Inventory-Human Services Survey* (MBI-HSS) para identificar a síndrome, com base nos critérios de Maslach e Grunfeld. A taxa de prevalência foi estimada juntamente do intervalo de confiança de 95% para cada dimensão da síndrome. As associações foram estimadas pela razão de chance (*odds ratio*), por meio de análises de regressão logística múltipla (α = 5%).

**Resultados:**

A prevalência da síndrome de *burnout* foi de 0,41% (0,01 - 2,29) segundo Maslach e 36,9% (30,82 - 43,36) segundo Grunfeld. Os profissionais das unidades de atendimento pediátrico tiveram maior probabilidade de desenvolver exaustão emocional (OR = 3,16). Aqueles com idade superior a 35 anos tiveram menos propensão a desenvolver exaustão emocional (OR = 0,32) e despersonalização (OR = 0,06). Maior número de horas de trabalho em unidades de terapia intensiva associou-se com diminuição da realização pessoal (OR = 1,13). Dentre os enfermeiros, os homens foram mais propensos à diminuição da realização profissional, e a ausência da prática de atividades físicas regulares associou-se com mais exaustão emocional e menos despersonalização. Dentre os médicos, o trabalho nas unidades de terapia intensiva pediátrica e cardiológica os tornou menos propensos a perceberem menor realização pessoal, e os médicos sem pós-graduação em terapia intensiva tiveram maior chance de apresentar sentimento de falta de realização pessoal.

**Conclusão:**

Este estudo demonstrou baixa prevalência da síndrome de *burnout*. Para cada dimensão de burnout, a maioria dos profissionais demonstrou baixos níveis de exaustão emocional, despersonalização e sentimento de falta de realização pessoal. Enfermeiros e médicos apresentaram diferentes características associadas com a síndrome de *burnout*.

## INTRODUCTION

Maslach et al.^(1)^ conceptualized Burnout syndrome as a multidimensional disease consisting of emotional exhaustion, dehumanization (depersonalization), and low personal accomplishment related to work. People cannot adequately deal with the chronification of work-related stress, and Burnout syndrome occurs when there is a lack of coping strategies, among other factors.

Emotional exhaustion refers to fatigue or the feeling of physical and mental exhaustion that prevents an individual from carrying out a task. Depersonalization includes negative behaviors and attitudes (coldness, excessive distancing, and indifference) towards service recipients among professionals. Finally, reduced personal accomplishment refers to professionals’ dissatisfaction with their activities, which reveals low professional efficiency.^(1-3)^

The prevalence of Burnout syndrome has increased over the last few years. ^(4)^ This tendency might be due to cold, competitive, hostile, and highly demanding work environments, such as intensive care units (ICU), which are known to be very stressful for both patients and their families.^(1,5)^

It is especially important to analyze the experience of Burnout syndrome among healthcare professionals who deal directly with critically ill patients, patients’ relatives, emergency situations and death to increase knowledge regarding occupational risks. However, most Brazilian studies on the subject have been based on small and convenient samples,^(6)^ which may have generated biased estimates of prevalence rates, as well as of the factors associated with the syndrome. Moreover, few studies have conducted analyses after adjusting for potential confounding variables, such as the context of labor, working process and social issues.^(7)^ Furthermore, the criteria for defining Burnout syndrome are not always explicit, nor is there any consensus regarding the cutoff points for its classification,^(8)^ which makes it difficult to compare estimates. However, there are models that present the development and diagnosis of burnout syndrome from various perspectives. The Grunfeld et al. model was chosen for the screening of Burnout syndrome in this study. This model proposes that burnout syndrome is present when there is a high level of emotional exhaustion, a high level of depersonalization or a low level of personal accomplishment.^(9)^

This study aimed to assess the prevalence of and factors associated with Burnout syndrome in ICU professionals.

## METHODS

This cross-sectional population-based study was conducted with all nurses and physicians from the 17 public ICU in São Luís, Maranhão, Brazil. Eight of these facilities are general ICU, four are neonatal ICU, two are infant, two are cardiologic ICU, and one is an oncologic ICU. This study was conducted from November 2011 to June 2013. The ethics committee of the *Universidade Federal do Maranhão* approved this study (protocol number: 006168/2009-70). We only included people who signed the informed consent form.

The inclusion criteria were being a nurse or physician employed by a hospital working in ICU patient care for more than one year. We did not include those who were on vacation or who took sick leave during the data collection period. Initially, 274 professionals met the criteria; however, 33 refused to participate, leaving a total of 241 professionals, including 125 nurses and 116 physicians.

We used a questionnaire to collect sociodemographic, cultural, and occupational data, and we used the Maslach Burnout Inventory - Human Services Survey (MBI-HSS) to diagnose Burnout syndrome.

The MBI-HSS is one of the best known instruments for research across different professional categories. The Burnout Inventory was translated to Portuguese and validated in 1997, yielding a Cronbach’s alpha of 0.86 for the emotional distress subscale, 0.69 for the depersonalization subscale and 0.76 for the personal accomplishment subscale.^(10)^

The questionnaire, which was created specifically for this survey, contained 17 questions distributed in the following blocks:

- Sociodemographic: gender (male or female), age (young adult, 18 - 35 years old; adult, 36 - 59 years old; elderly, 60 years or older), marital status (with a partner or not with a partner), religion (yes or no), professional category (physician or nurse), postgraduate degree (yes or no), and postgraduate degree related to ICU (yes or no).
- Occupational: length of professional experience (computed as the length of time between graduation and the interview date, categorized according to the distribution tertiles as follows: < 4 years, 4 - 14 years, or ≥ 15 years, for nurses, and ≤ 7 years, 8 - 18 years, or ≥ 19 years, for physicians); ICU specialization (infant, including patients from the neonatal and pediatric age group; general and cardiology); length of time working in an ICU (categorized based on the distribution tertiles as: ≤ 2 years, 3 - 9 years or ≥ 10 years for nurses and ≤ 3 years, 4 - 13 years or ≥ 14 years for physicians); hourly workload (≤ 40 hours and > 40 hours); and location of the majority of ICU work (public or private).
- Habits: exercise (yes or no); exercise frequency (zero to 2 times, irregular; and more than 3 times, regular); current use of any type of alcoholic beverage (yes or no).
- Cultural: importance of religion (very important or very little/not important); preferred leisure activity (movies/theater: yes or no; concerts: yes or no; happy hour: yes or no; soccer: yes or no; and others: yes or no).

The MBI-HSS is a self-report instrument created by Maslach and Jackson. In its current version, the MBI-HSS consists of 22 Likert-type questions with 7 response options, ranging from 0 (never) to 6 (daily). The MBI-HSS is divided into three subscales corresponding to three dimensions that evaluate the possible manifestations of Burnout syndrome: “emotional exhaustion”, “depersonalization”, and “reduced personal accomplishment” (the latter dimension is inversely related to the syndrome). The first dimension has nine items, the second has five items, and the third has eight items.^(11)^

The MBI-HSS scale has already been translated, adapted, and validated for use in Brazilian studies by multiple authors. Lautert’s^(12)^ validation was chosen because it was applied to participants similar to those in this study.

In terms of quantifying the scores, no consensus currently exists for diagnosing Burnout syndrome using the values obtained in the MBI-HSS. Therefore, most authors only describe the percentage results or the mean and standard deviations in the three dimensions to define the syndrome. In our study, we categorized each of the three dimensions of the syndrome into low, average and high risk. However, in the regression analyses, we presented only comparisons between high *versus* low risk, which allowed us to compare the categories of extreme risk and to identify variables associated with the occurrence of burnout in its most severe form. The evaluation proposed by the inventory itself states that the syndrome is defined when an individual presents a change in the three dimensions.^(11)^ Grunfeld et al.^(9)^ characterized Burnout syndrome as the presence of a change in only one dimension of burnout, that is, either high levels of emotional exhaustion, high levels of depersonalization, or low levels of Personal Accomplishment. We verified the results of all the evaluations of Burnout syndrome.

Statistical analysis was performed using the STATA program, version 11. The Shapiro-Wilk test was used to evaluate the data distribution. Variables were summarized using the mean ± standard deviation (SD), and the qualitative variables were summarized through absolute (n) and percentage (%) values. The prevalence rates of the syndrome, according to their dimensions, were estimated at 95% confidence intervals (95%CI). We weighted all the analyses for the unequal probabilities of participant selection using the commands *svy*. This strategy is useful for adjusting estimates for potential selection biases.

Chi-squared and Fisher’s exact tests were used for comparisons of categorical dependent variables. The associations were estimated via odds ratios (OR) and their respective 95%CI in multiple logistic regression analyses. The associations were estimated for all the professionals as well as for the nurses and physicians independently.

A stepwise modeling strategy was used, and a significance level of 10% (alpha = 0.10) was adopted as a criterion for selecting the variables to be included in the adjusted model. A significance level of 5% was adopted in all the analyses as a criterion for rejecting the null hypothesis (H0).

## RESULTS

We interviewed 241 ICU workers. More than half (50.7%) of the ICU workers were nurses and 49.3% were physicians. The prevalence of Burnout syndrome, according to the criteria of Maslach et al.,^(11)^ was 0.41% (95%CI 0.01 - 2.29), and according to the criteria of Grunfeld et al.,^(9)^ it was 36.9% (95%CI 30.82 - 43.36). Separating the results by the syndrome-defining dimensions, 28.9% (95%CI 23.01 - 34.78) of the participants experienced high emotional exhaustion, 6.3% (95%CI 3.52 - 10.06) experienced high depersonalization levels, and 10.9% (95%CI 7.16 - 15.41) had very low personal accomplishment.

The nurses’ mean age was 36.5 (± 8.2) years, and their mean duration of working in an ICU was 6.8 (± 5.8) years. For the physicians, their mean age was 38.5 (± 8.3) years, and they had spent an average of 8.3 (± 6.5) years working in an ICU.

Both the nurses and physicians were primarily female (59.4% and 87.2%), lived with a partner (50.4% and 72.4%), were religious (97.6% and 92.2%), and assigned great importance to their religious beliefs (95.2% and 89.6%). In addition, both the nurses and physicians mostly worked in a general (45.6% and 39.6%) or infant (37.6% and 41.3%) ICU and had completed a graduate degree (85.2% and 91.3%) but did not have a postgraduate degree related to ICUs (53.3% and 59.1%). Current alcohol consumption was observed in 20.5% of the nurses and 25.3% of the physicians. Only 16% of the nurses exercised regularly, but 51.7% (p < 0.05) of the physicians exercised (Table 1).

**Table 1:** Sociodemographic, behavioral and occupational data of public intensive care unit professionals

	All professionals*	Nurses*	Physicians*	p value*^†^
Covariables				
Sex				< 0.001
Male	63 (21.3)	16 (9.4)	47 (43.1)	
Female	178 (78.7)	109 (90.6)	69 (56.9)	
Age (years)				0.580
≤ 35	97 (44.8)	54 (47.2)	43 (40.6)	
≥ 36	144 (55.2)	71 (52.8)	73 (59.4)	
Marital status				0.003
With a partner	147 (57.1)	63 (46.3)	84 (76.8)	
Not with a partner	94 (42.9)	62 (53.7)	32 (23.2)	
Religion				0.005
Yes	229 (96.6)	122 (98.8)	107 (92.7)	
No	12 (3.4)	3 (1.2)	9 (7.3)	
Importance of religion				0.007
Very important	223 (95.5)	119 (97.8)	104 (91.2)	
Not very/not important	18 (4.5)	6 (2.2)	12 (8.8)	
ICU specialization				0.529
General	108 (59.7)	59 (62.7)	49 (54.1)	
Cardiology	29 (12.5)	15 (12.7)	14 (12.2)	
Infant	95 (27.8)	47 (24.6)	48 (37.7)	
Exercise regularly				0.241
Yes	80 (34.0)	20 (27.9)	60 (45.1)	
No	161 (66.0)	105 (72.1)	56 (54.9)	
Postgraduate degree				0.556
Yes	210 (91.0)	104 (90.0)	106 (92.8)	
No	28 (9.0)	18 (10.0)	10 (7.2)	
Postgraduate degree related to ICU				0.355
Yes	92 (53.7)	49 (57.8)	43 (46.5)	
No	118 (46.3)	56 (42.2)	62 (53.5)	
Burnout dimensions				
Emotional exhaustion				0.185
Low	94 (38.3)	39 (33.1)	55 (47.6)	
Average	75 (35.3)	45 (41.9)	30 (23.4)	
High	69 (26.4)	38 (25.0)	31 (29.0)	
Depersonalization				0.120
Low	172 (70.2)	91 (69.2)	81 (72.1)	
Average	51 (14.6)	24 (10.8)	27 (21.4)	
High	15 (15.2)	7 (20.0)	8 (6.5)	
Reduced personal accomplishment				0.371
Low	137 (55.1)	66 (53.2)	71 (58.5)	
Average	75 (34.7)	41 (39.0)	34 (27.0)	
High	26 (10.2)	15 (7.8)	11 (14.5)	
Burnout syndrome				
Maslach	1 (0.2)	0 (0.0)	1 (0.6)	0.182
Grunfeld	89 (43.4)	49 (44.7)	40 (41.0)	0.766

ICU - intensive care unit. Results expressed as n (%).

* Weighted for the unequal probabilities of participant selection

† Chi-squared.

Changes in the three dimensions were not observed among the nurses working in public ICU in São Luís, Brazil. However, according to the criteria of Grunfeld, 39.2% of the nurses presented with Burnout syndrome, 31.1% of them had high levels of emotional exhaustion, 5.7% had high levels of depersonalization, and 12.3% were not satisfied with their work. Among the physicians, the prevalence of Burnout syndrome was 0.86% according to Maslach’s definition and 34.4% according to Grunfeld. For each dimension of Burnout syndrome, 26.7% of the physicians had high levels of emotional exhaustion, 6.9% had high levels of depersonalization, and 9.48% had low personal accomplishment.

Since no cases satisfied the diagnostic criteria for Burnout syndrome according to Maslach’s criteria for nurses, the modeling of this outcome was not possible. However, we could estimate the associations of the sociodemographic, behavioral, and cultural data with each of the three domains of Burnout syndrome separately.

Among nurses, being male increased the odds of a reduced sense of personal accomplishment (OR = 9.71). Being older than 35 years of age reduced the chances of developing emotional exhaustion (OR = 0.08) and depersonalization (OR = 0.02). Not exercising regularly increased the chance of developing a high level of emotional exhaustion (OR = 11.01) and reduced the chance of depersonalization (OR = 0.07). The reduced sense of personal accomplishment was more likely among nurses working in an infant ICU (OR = 4.74) than among nurses working in a general ICU (Table 2).

**Table 2:** Covariables associated with each dimension of burnout among nurses

Covariables	Emotional exhaustion (high versus low level)						Depersonalization (high versus low level)						Reduced personal accomplishment (high versus low level)					
	Crude			Adjusted			Crude			Adjusted			Crude			Adjusted		
	OR	95%CI	p value	OR	95%CI	p value	OR	95%CI	p value	OR	95%CI	p value	OR	95%CI	p value	OR	95%CI	p value
Male gender	1.71	0.29 - 10.01	0.544				1.71	0.11 - 25.90	0.698				8.39	1.27 - 55.23	0.027	9.71	1.96 - 47.98	0.006
Age > 35 years old	0.16	0.04 - 0.60	0.007	0.08	0.02 - 0.36	0.001	0.01	0.001 - 0.14	< 0.001	0.02	0.002 - 0.21	0.001	0.59	0.11 - 3.11	0.533			
Lives with a partner	1.26	0.29 - 5.46	0.751				7.98	0.66 - 96.56	0.101				0.97	0.22 - 4.35	0.967			
Length of time working in an ICU	0.93	0.80 - 1.07	0.285				0.74	0.56 - 0.98	0.035				1.11	0.93 - 1.32	0.237			
Does not exercise regularly	7.36	1.14 - 47.32	0.036	11.01	2.73 - 44.39	0.001	0.05	0.004 - 0.61	0.019	0.07	0.007 - 0.79	0.032	0.49	0.07 - 3.52	0.473			
Hourly workload in ICU	0.98	0.95 - 1.03	0.598				1.00	0.96 - 1.05	0.948				0.98	0.94 - 1.02	0.280			
Cardiology ICU*	1.63	0.23 - 11.52	0.619				0.53	0.02 - 9.47	0.661				4.40	0.37 - 51.91	0.235	1.20	0.14 - 10.52	0.867
Infant ICU*	3.79	0.96 - 15.02	0.058				0.07	0.006 - 0.80	0.033				5.89	1.25 - 27.68	0.025	4.73	1.10 - 20.24	0.036
No postgraduate degree related to ICU	0.76	0.15 - 3.86	0.735				0.07	0.007 - 0.69	0.024				1.09	0.17 - 6.83	0.926			
Length of professional experience	--						0.82	0.68 - 0.99	0.041				1.06	0.97 - 1.17	0.189			

OR - odds ratio; 95%CI - 95% confidence interval; ICU - intensive care unit.

* Compared to general intensive care unit.

Physicians who worked in a cardiology (OR = 0.04) or infant (OR = 0.11) ICU had a lower chance of having a reduced sense of personal accomplishment. Physicians without an ICU specialization had a higher chance of having a reduced sense of personal accomplishment (OR = 17.54). Longer professional experience was associated with a lower chance of depersonalization (OR = 0.89) (Table 3).

**Table 3:** Covariables associated with each dimension of burnout among physicians

Covariables	Emotional exhaustion (high versus low level)						Reduced personal accomplishment (high versus low level)						Depersonalization (high versus low level)					
	Crude			Adjusted			Crude			Adjusted			Crude			Adjusted		
	OR	95%CI	p value	OR	95%CI	p value	OR	95%CI	p value	OR	95%CI	p value	OR	95%CI	p value	OR	95%CI	p value
Male gender	0.82	0.23 - 2.96	0.760				4.75	0.91 - 24.69	0.063	3.42	0.65 - 18.11	0.146	0.09	0.01 - 0.92	0.043	--		
Age > 35 years old	1.00	0.28 - 3.64	0.996				0.64	0.12 - 3.48	0.604				4.14	0.66 - 25.82	0.127	--		
Lives with a partner	0.94	0.29 - 3.04	0.917				0.36	0.04 - 3.42	0.371				1.08	0.19 - 5.98	0.930			
Length of time working in an ICU	0.94	0.87 - 1.03	0.176	0.94	0.86 - 1.02	0.142	0.96	0.86 - 1.06	0.434				1.14	1.00 - 1.30	0.051	--		
Does not exercise regularly	1.42	0.45 - 4.49	0.541				0.83	0.15 - 4.54	0.832				2.88	0.55 - 15.08	0.206			
Hourly workload in ICU	1.01	0.98 - 1.04	0.572				1.01	0.95 - 1.07	0.788				0.98	0.95 - 1.02	0.435			
Cardiology ICU*	0.12	0.01 - 1.33	0.085	0.11	0.01 - 1.30	0.080	4.83	0.59 - 39.39	0.139	7.69	0.83 - 70.98	0.072	0.15	0.01 - 1.69	0.123	0.04	0.01 - 0.64	0.023
Infant ICU*	1.82	0.50 - 6.60	0.354	1.97	0.55 - 7.08	0.296	0.95	0.11 - 8.10	0.961	2.90	0.30 - 28.35	0.355	0.49	0.10 - 2.39	0.373	0.11	0.01 - 0.87	0.037
No postgraduate degree related to ICU	0.61	0.18 - 2.16	0.444				--						4.61	0.66 - 32.27	0.121	17.54	1.59 - 92.94	0.020
Length of professional experience	0.97	0.90 - 1.04	0.377				0.94	0.88 - 1.00	0.061	0.89	0.80 - 0.98	0.028	1.09	0.97 - 1.23	0.129	--		

OR - odds ratio; 95%CI - 95% confidence interval; ICU - intensive care unit.

* Compared to general intensive care unit.

Considering both professions jointly, being older than 35 years of age decreased the likelihood of developing emotional exhaustion (OR = 0.32) and depersonalization (OR = 0.06). There was a higher chance of having a reduced sense of professional accomplishment (OR = 1.13) among professionals with a longer length of time working in an ICU (OR = 1.13). The professionals who did not exercise had a lower chance of depersonalization (OR = 0.14). Those who worked in the infant ICU were more likely to experience emotional exhaustion (OR = 3.16) (Table 4).

**Table 4:** Covariables associated with each dimension of burnout among nurses and physicians jointly

Covariables	Emotional exhaustion (high versus low level)						Depersonalization (high versus low level)						Reduced personal accomplishment (high versus low level)					
	Crude			Adjusted			Crude			Adjusted			Crude			Adjusted		
	OR	95%CI	p value	OR	95%CI	p value	OR	95%CI	p value	OR	95%CI	p value	OR	95%CI	p value	OR	95%CI	p value
Male gender	1.02	0.36 - 2.86	0.973				1.22	1.16 - 9.10	0.844				1.05	0.24 - 4.54	0.949			
Age > 35 years old	0.38	0.15 - 0.94	0.036	0.32	0.12 - 0.85	0.023	0.06	0.01 - 0.36	0.003	0.06	0.01 - 0.37	0.002	1.40	0.41 - 4.66	0.585			
Lives with a partner	1.16	0.44 - 3.08	0.757				5.21	0.69 - 39.15	0.108	--			0.96	0.31 - 3.01	0.944			
Length of time working in an ICU	0.93	0.86 - 1.01	0.093	--			0.82	0.68 - 0.99	0.047	--			1.13	1.02 - 1.25	0.018	1.13	1.02 - 1.25	0.018
Does not exercise regularly	2.45	0.92 - 6.54	0.072	--			0.12	0.02 - 0.93	0.043	0.14	0.02 - 0.99	0.049	1.26	0.39 - 4.09	0.696			
Hourly workload in ICU	0.99	0.97 - 1.03	0.800				1.01	0.97 - 1.04	0.655				0.98	0.95 - 1.01	0.130	--		
Cardiology ICU*	0.84	0.18 - 3.85	0.822	0.66	0.17 - 2.63	0.554	0.75	0.07 - 7.53	0.810	--			0.99	0.12 - 8.05	0.998			
Infant ICU*	2.71	1.03 - 7.08	0.043	3.16	1.19 - 8.42	0.021	0.12	0.01 - 0.93	0.043	--			1.55	0.44 - 5.42	0.491			
No postgraduate degree related to ICU	0.71	0.25 - 2.02	0.523				0.22	0.03 - 1.49	0.121	--			2.08	0.52 - 8.31	0.299			
Length of professional experience	0.96	0.91 - 1.02	0.222				0.85	0.73 - 0.99	0.040	--			1.08	1.00 - 1.16	0.036	--		

OR - odds ratio; 95%CI - 95% confidence interval; ICU - intensive care unit.

* Compared to general intensive care unit.

## DISCUSSION

We found a prevalence of Burnout syndrome of 0.41% according to Maslach’s criteria and of 36.9% according to Grunfeld’s criteria. Infant ICU professionals were more likely to develop emotional exhaustion than other ICU professionals, while those over 35 years of age were less likely to develop emotional exhaustion and depersonalization. Longer working hours in an ICU were associated with a reduced sense personal accomplishment.

In Romania, among 146 physicians, 30% had Burnout syndrome according to Maslach’s criteria,^(13)^ and in Spain, there is a high prevalence of burnout among ICU nurses.^(14)^ However, in most Brazilian studies, the reality is quite different, and the prevalences vary among regions. The prevalence of Burnout syndrome varied from zero among 151 nurses at a large hospital in southern Brazil^(15)^ to 31.5% among 178 ICU professionals throughout the country.^(16)^

Studies that evaluated the syndrome based on Grunfeld’s criteria reported higher rates of Burnout syndrome than those using Maslach’s criteria. This difference was also found in the present study, with a prevalence of 39.2% among nurses and 34.4% among physicians. In France, 45.6% of 978 physicians had Burnout syndrome,^(17)^ and in the United States, half of physicians reported experiencing Burnout in 2014. ^(4)^ In northeastern Brazil, 63.3% of intensivist physicians in the state of Bahia^(18)^ and 70% of ICU physicians in Maceió^(19)^ had Burnout syndrome. In the south, the prevalence of Burnout syndrome among nursing staff was 35.7%,^(15)^ similar to the results in our research.

Nurses older than 35 years of age had lower chances of developing emotional exhaustion and depersonalization. Lin et al.^(20)^ found similar results by indicating that Burnout syndrome was more prominent among younger professionals. Gomez-Urquiza et al.^(21)^ also found an association between young nurses and depersonalization in Spain. Most likely, more experienced nurses are more capable of dealing with work demands.

Nurses who do not exercise regularly were more likely to develop emotional exhaustion and less likely to exhibit depersonalization, similar to the findings from other studies.^(22-24)^ Physical activity is one of the most commonly used strategies for coping with Burnout syndrome because it promotes benefits for the body, controls stress and reduces levels of anxiety and depression.^(25)^ On the other hand, physical activity was associated with depersonalization. It is possible that professionals who frequently use fitness spaces, such as gyms, parks, and beaches, deal directly with people focused on health and wellness; these professionals are then overburdened when treating very ill people and try to preserve a relationship not focused on the imminence of death.

A reduced sense of professional accomplishment was more likely to be present among nurses working in infant ICU than among professionals working in other types of ICU. Although there are discrepancies between different cultures, society in general presents difficulties in dealing with hospitalization and the death of children. The interruption of an infant’s life generates negative emotions and is considered tragic, unfair and unnatural.^(26)^ The illness or death of children can go against the professional’s personal values, thus generating a greater sense of failure.^(27)^

Unlike nurses, physicians working in the infant and cardiology ICU were less likely to feel reduced personal accomplishment. However, a Brazilian study has shown the opposite association - that the work of physicians in the pediatric ICU is related to a higher occurrence of Burnout syndrome.^(28)^ This contradiction might be justified by the particularity of the work in different Brazilian public services. Pediatric and cardiology ICU in the city of São Luís are linked to the Public Health System and to the University Hospital, and because they are complex hospital units, they potentially generate feelings of comfort, safety and autonomy for labor actions among physicians. Although they deal with death, these professionals are capable of saving lives, including those of children, which can give meaning to their work and increase their sense of personal accomplishment.

In our study, physicians with no postgraduate degree who worked in an ICU had a higher chance of having a reduced sense of personal accomplishment. A greater probability of Burnout syndrome was also observed in intensivists with less than nine years of ICU training^(18)^ and with no ICU specialization.^(28)^ These physicians had more physical and psychological exhaustion related to work.^(28)^ In Brazil, medical schools prioritize the biomedical model and invest little in palliative care. Health workers usually think about the death of patients only when they are already working. Moreover, death is a taboo in Western society and is often understood as a failure in the hospital field.

Colleges still offer technical training, without any preparation for situations of loss, death and mourning.^(29)^ Because the ICU environment includes patients with diseases of high complexity and with the possibility of impending death, it generates investments in technological equipment that can deal with diseases to control the human body.^(30)^ Operating such devices requires more specializations and additional hours of study beyond those already experienced in undergraduate and residency. It is therefore possible that there is a greater level of insecurity for those who do not yet have such knowledge, which can lead to feelings of incompetence and less personal accomplishment.

A longer professional experience was associated with lower chances of depersonalization. In our research, physicians had longer professional experience (average = 13.4 years) than nurses. Older and more experienced workers are more resilient, while young workers with high job tenures have particular vulnerability and a lack of resources that can help in the execution of their job demands.^(31)^ Therefore, having more professional experience would mean more maturity, thus averting the possibility of cold and negative attitudes towards their patients.

Some issues can be raised regarding the low prevalence of Burnout syndrome. Was the MBI-HSS unable to evaluate the population of nurses and physicians who work in the ICU in the city of São Luís? Or were the coping strategies used by the professionals to confront the syndrome effective?

Coping strategies may be a response to the low prevalence of burnout since they are used to defend the ego and help people deal with disagreeable emotions and stressful events. The MBI-HSS, despite being the most widely used international instrument for measuring Burnout syndrome, presents divergences with regard to its evaluation, especially due to its cross-cultural instability.^(32)^ In Brazil, the MBI-HSS shows an average consistency in terms of its depersonalization subscale, in addition to facing criticism related to depersonalization and the formulation of items present on the inventory.^(33)^

Some issues such as “*feel like you are treating people like inanimate objects*” may shock health professionals due to their indifference and emotional rigidity. These professionals, influenced by social and religious rules, end up responding to the inventory in accordance with the way they believe they should act,^(33)^ rather than the behavior they exhibit every day when caring for patients. In addition, the MBI-HSS does not indicate the presence of all symptoms (physical, psychic, behavioral, and defensive) that determine Burnout syndrome.

As a way to overcome these difficulties, many studies worldwide seek to complement the MBI-HSS by using other scales that assess stress, work perceptions, personality characteristics, health-related information, and sociodemographic data.^(2,22,31-33)^ Burnout levels are different from country to country. Additionally, explanations for these facts depend not only on the culture of each population but also on the variations between professional categories and labor concepts themselves.^(34)^ There is an understanding that each country needs its own inventory.^(9,33)^

We could not deeply investigate other sociodemographic and occupational variables that could help us to better understand Burnout syndrome or its dimensions. A perception of the variables associated with Burnout syndrome could guide the construction and validation of new instruments for diagnosing the syndrome and reduce the difficulties related to analyzing burnout.

## CONCLUSION

The prevalence of Burnout syndrome is low among physicians and nurses who work in public intensive care units in São Luís, Brazil. Most of the professionals reported low levels for each dimension of burnout, including low levels of emotional exhaustion, low levels of depersonalization, and a nonreduced sense of personal accomplishment. Nurses and physicians have different characteristics associated with Burnout syndrome.

Prevention and intervention should be thought of from three levels: programs centered on individual response, programs centered on organizational context, and programs centered on interaction between the organization and the individual context.

Psychoeducation about burnout, the adoption of healthy living habits, assertiveness training and communication skills, relaxation, social support and individual psychotherapy are strategies that can be used in programs focused on individual response. In organizational programs, the intervention should focus on environmental planning for work execution. On the third level, the intervention involves the combined use of both strategies. These programs have a preventive purpose if practiced continuously.

This article contributes to the understanding of Burnout syndrome among health workers. Identifying the personal, social, organizational and psychological factors associated with Burnout syndrome is important to the development of preventive actions for mental health.
